# The Satellite Cell in Male and Female, Developing and Adult Mouse Muscle: Distinct Stem Cells for Growth and Regeneration

**DOI:** 10.1371/journal.pone.0037950

**Published:** 2012-05-25

**Authors:** Alice Neal, Luisa Boldrin, Jennifer Elizabeth Morgan

**Affiliations:** 1 The Dubowitz Neuromuscular Centre, Institute of Child Health, University College London, London, United Kingdom; 2 MRC Centre for Neuromuscular Diseases, Institute of Neurology, University College London, London, United Kingdom; University of Pittsburgh, United States of America

## Abstract

Satellite cells are myogenic cells found between the basal lamina and the sarcolemma of the muscle fibre. Satellite cells are the source of new myofibres; as such, satellite cell transplantation holds promise as a treatment for muscular dystrophies. We have investigated age and sex differences between mouse satellite cells *in vitro* and assessed the importance of these factors as mediators of donor cell engraftment in an *in vivo* model of satellite cell transplantation. We found that satellite cell numbers are increased in growing compared to adult and in male compared to female adult mice. We saw no difference in the expression of the myogenic regulatory factors between male and female mice, but distinct profiles were observed according to developmental stage. We show that, in contrast to adult mice, the majority of satellite cells from two week old mice are proliferating to facilitate myofibre growth; however a small proportion of these cells are quiescent and not contributing to this growth programme. Despite observed changes in satellite cell populations, there is no difference in engraftment efficiency either between satellite cells derived from adult or pre-weaned donor mice, male or female donor cells, or between male and female host muscle environments. We suggest there exist two distinct satellite cell populations: one for muscle growth and maintenance and one for muscle regeneration.

## Introduction

The satellite cell is the principal muscle stem cell [1]–[3]. Satellite cells, found between the basal lamina and the sarcolemma of the muscle fibre, proliferate to produce a pool of mononucleated cells that fuse together to become multinucleated myofibres [4]–[6]. As the source of myofibres, satellite cells are a promising target for the development of stem cell therapies for muscular dystrophies. Satellite cell transplantation can successfully restore dystrophin expression in the immunodeficient mouse model of Duchenne muscular dystrophy, the *mdx*-nude [7], [8], at any age [9], [10]. However, not all satellite cells have the same regenerative capacity and many do not survive transplantation [7]. Thus, it is necessary to investigate properties of satellite cells that may mediate proliferation and survival, and factors within the host environment that are conducive to greater donor satellite cell contribution to muscle regeneration post transplantation.

Males have greater muscle weights and larger skeletal muscle cross sectional areas than females [11], [12]. This is in part mediated by exposure to circulating androgens [13]. Androgens have been shown to have a direct influence on satellite cell proliferation and differentiation [14], [15]. For example, testosterone treatment has been associated with an increase in myonuclei and satellite cell number in hypogonadal men and with the induction of DNA synthesis in the *levator ani* muscle of the rat [16], [17]. In addition to androgen-mediated changes in their activity, there are some data to suggest that male and female satellite cells are intrinsically distinct [18], [19]. However, to properly assess differences between satellite cell populations, it is necessary to consider that satellite cells are responsible for both muscle growth during early postnatal life [20]–[22] and for muscle regeneration. Both processes involve fusion of satellite or daughter cells either with the fibre (during growth or repair of segmental necrosis [23]), or with each other to form entirely new myofibres, during regeneration following extensive injury. Regeneration is characterized by necrosis of damaged tissue, activation of an inflammatory response and the activation of quiescent satellite cells to repair/replace fibres in response to muscle damage (reviewed [24], [25]), whereas signals mediating muscle growth appear to emanate from the fibre itself [26], [27]. It is highly likely that, as these processes have differing initiating signals, they also have differing regulatory molecular mechanisms [26], [28], [29]. The relative efficiency of these processes may be considerably influenced by the developmental stage of the muscle from which these satellite cells originate.

The role of the satellite cell in adult muscle maintenance is the source of much debate [30]–[35]. The myonuclear domain hypothesis states that each myofibre maintains a constant ratio of myonuclei to cytoplasm and stem cell activity is therefore required for any increase in myofibre size [36]. However, the myonuclear domain hypothesis is challenged by studies that show changes in muscle fibre size but not in myonuclear number [37]–[39]. Either satellite cells are required for muscle growth and regulation of myofibre size and thus constitute a population of cells that regularly enters the proliferative state, or they are a population of cells that are in deep quiescence for the majority of their existence, becoming activated only upon muscle injury.

Although the role of the satellite cell in healthy adult skeletal muscle remains ambiguous, the satellite cell occupies a relatively undisputed position as a cell that is essential for muscle regeneration [1], [7]. The relative efficiency of male and female satellite cells to regenerate dystrophic muscle remains unexplored. Considering the previously mentioned influence of circulating androgens on satellite cell activity, it is likely that the host environment will mediate donor cell engraftment efficiency. In addition, if there are indeed intrinsic differences in male and female satellite cells, the sex from which donor satellite cells are derived may also affect the extent of donor cell contribution to regeneration.

The efficiency of skeletal muscle regeneration is influenced by the developmental stage of the muscle. Aged muscles show a decrease in regenerative capacity compared to adult, which is widely considered to be environmentally regulated [40], [41]. In the mouse, satellite cells from growing muscles have been shown to have distinct genetic requirements compared to those from the adult [28]. Satellite cell expression of Pax7 is critical for satellite cell specification and muscle regeneration before postnatal day 21, but, after this critical time period, satellite cells are able to mount an efficient regenerative response in the absence of Pax7 [28]. Muscle precursor cells derived from muscle of young and newborn mice are able to regenerate adult host muscle post transplantation [42]–[44], but whether these cells, derived from enzymatic disaggregation of muscle, are actually satellite cells or another cell type (e.g. PICS, present in large numbers in newborn mouse muscle [45]) was not ascertained. The post transplantation regenerative capacity of a pure satellite cell population, derived from fibres of young actively growing mice is unknown. There has been no systematic, quantitative comparison of the regenerative capacity of young actively growing compared to adult satellite cells.

Here, we have characterised the male and female satellite cell populations of the C57BL/6 mouse across its lifespan both *in vitro* and *in vivo*. We show that adult males have more satellite cells per fibre than adult females. In accordance with previous reports [7], [46]–[50], we show that the number of satellite cells per fibre is significantly increased in growing compared to adult and in adult compared to aged fibres. A correlation between myofibre volume and myonuclear number is observed, but exclusively in adult mice. From this we conclude that muscle maintenance requires satellite cell activity. Despite differences in absolute satellite cell number, *in vitro* myogenic regulatory factor expression profiles and proliferative rates, growing and adult, male and female satellite cells show comparable regenerative capacity post transplantation. These data provide evidence for the existence of at least two distinct populations of satellite cells. The first is responsible for muscle growth and routine muscle maintenance; this population decreases with age and is reduced in female compared to male mice. The second population is activated only in response to muscle injury, survives transplantation and its numbers are not altered by sex or age.

## Results

### Satellite cell numbers decline with age and are greater in male compared to female adult mice

**Figure 1:**
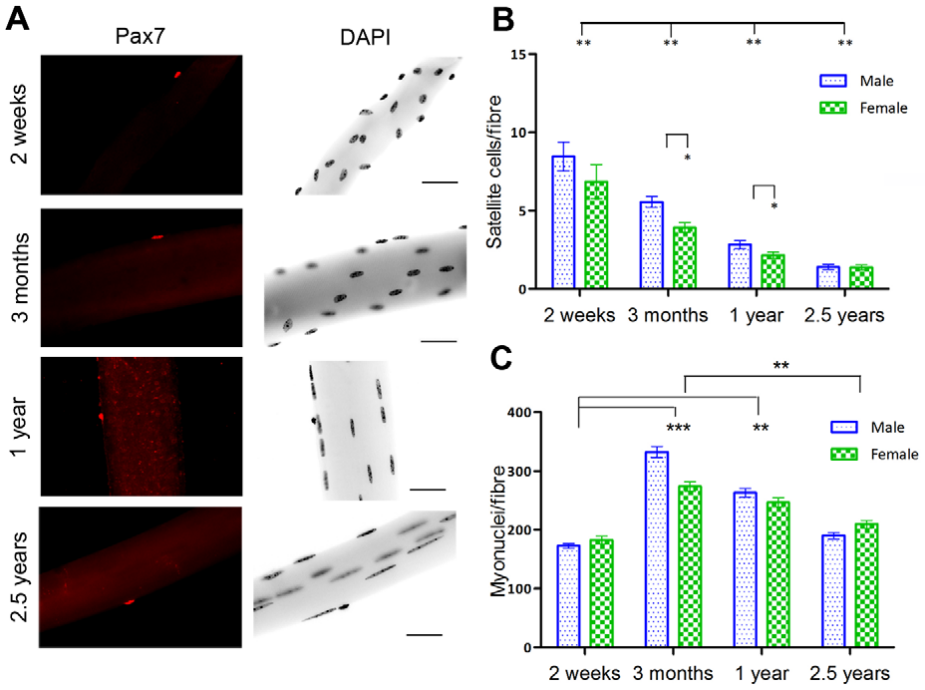
Satellite cell and myofibre number per EDL myofibre in male and female mice across the lifespan. A) Isolated single fibres immunostained with Pax7 (i) and DAPI (ii) show myonuclei and satellite cells. Scale bar 50 µm. (B) 2 way analysis of variance test revealed a significant main effect of both age (p<0.0001) and gender (p = 0.003). Satellite cell numbers decline steadily with age: 2 week old mice have significantly more satellite cells per fibre than any other age group (8.5+/−0.93, 6.5+/−1.10 satellite cells/fibre in males and females respectively). Post hoc t tests show satellite cell numbers declined significantly between 2 weeks and 3 months (p = 0.03) 3 months and 1 year (p<0.001) and declined still further between 1 and 2.5 years (p<0.0001). Males were shown to have significantly greater numbers of satellite cells per fibre than females at 3 months (p = 0.02) and 1 year (p = 0.03) of age. At 2 weeks and 2.5 years, there was no difference between male and female satellite cell numbers per fibre. (C) 2 way analysis of variance test showed a significant effect of age (p<0.0001) and gender (p = 0.04) and an interaction between these variables (p<0.0001) on the number of myonuclei per fibre. The number of myonuclei per fibre increased significantly between 2 weeks and 3 months of age (p = 0.001) but showed a significant decline between 1 year and 2.5 years (p<0.001). Males had significantly more myonuclei per fibre than females at 3 months but at no other age investigated. Data show mean and s.e.m.

To begin our understanding of age and sex differences in satellite cell populations, we quantified satellite cell numbers on freshly-isolated myofibres derived from the *extensor digitorum longus* (EDL) muscle of 3 month, 1 year and 2.5 year old male and female C57BL/6 mice. Satellite cells were quantified using immunostaining for Pax7 and co-localisation with DAPI on fixed single fibres (Fig. 1A). The number of Pax7 expressing cells was at its highest at 2 weeks of age (8.5+/−0.9, 6.5+/−1.0 satellite cells/fibre respectively in males and females) and declined steadily thereafter, reaching a low of just 1.4+/−0.1 satellite cells per fibre at 2.5 years of age in males (Fig. 1B). Each data point represents the mean of 60 fibres sampled from 3 mice. Males had significantly more satellite cells per fibre than females during the adult years (p = 0.02 at 3 months, p = 0.03 at 1 year).

To investigate the nature of the relationship between satellite cell and myonuclear number, we quantified the number of myonuclei per fibre on fibres isolated from male and female mice of different ages (Fig. 1C). Two week old mice had significantly less myonuclei per fibre than any other age group studied (173+/−30, 182+/−42 nuclei/fibre respectively in males and females). The decrease in satellite cell number observed between 3 month old and 2 week old mice coincided with an increase in myonuclear number (p<0.0001). From this we infer that many of the Pax7 expressing cells observed at 2 weeks of age undergo terminal differentiation without self-renewal and that the proliferation and differentiation of these cells is responsible for growth and the increase in myonuclear number.

Mirroring the drop in satellite cell numbers, myonuclear number was significantly reduced in 2.5 year old compared to 1 year old mice (p = 0.001). Although these data do not demonstrate a causative relationship, it is possible that the decrease in myonuclei is due to the inability of a diminished satellite cell pool to maintain nuclei number. This hypothesis is widely supported in the literature [47]–[51]

### Growing, adult and aged satellite cell populations have distinct myogenic regulatory factor expression profiles *in vitro*

Satellite cells express different combinations of the myogenic regulatory factors Pax7, MyoD and myogenin according to their activation, proliferation and differentiation status. Quiescent satellite cells express Pax7 but do not express MyoD. After 24 hours in culture, all satellite cells up regulate MyoD and enter into the proliferative cycle to produce colonies of myoblasts. After this proliferative phase, most satellite cells will go on to differentiate, during which they express myogenin and fuse, either to an existing fibre for repair and myonuclear addition, or to other myoblasts to form new fibres. However, a rare few satellite cells will down-regulate MyoD and retain Pax7. These cells represent a population of satellite cells that are returning to quiescence to provide a pool of satellite cells for future need [6]. The number of cells positive for Pax7 and negative for MyoD after 72 hours in culture is therefore a measure of satellite cell self-renewal.

**Figure 2:**
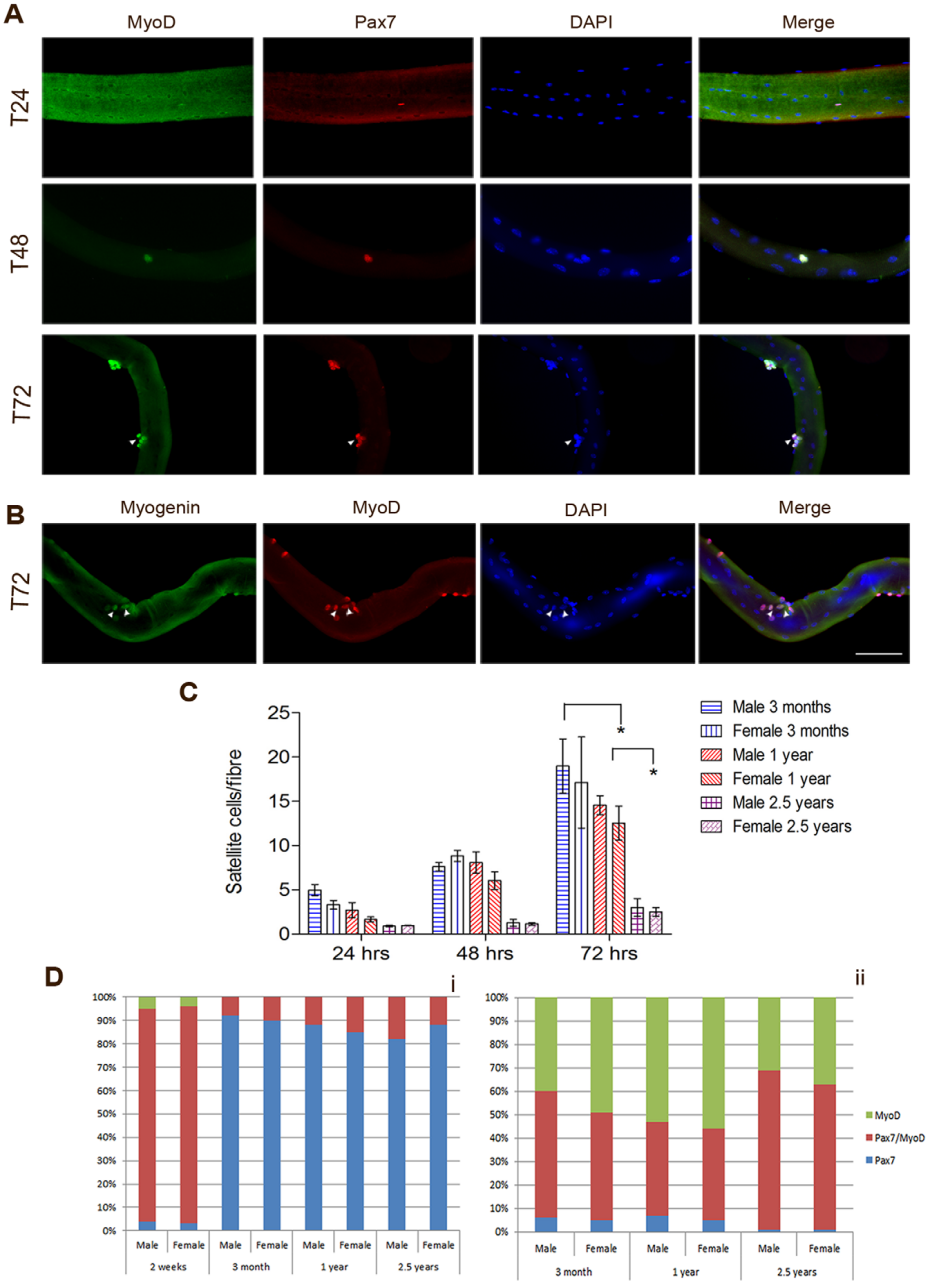
Satellite cell proliferation and self-renewal in male and female growing, adult and aged cultures. (A) Representative images of single fibres fixed after 24, 48 or 72 hours in suspension and immunostained for Pax7 and MyoD. After 72 hours individual satellite cells have up-regulated MyoD and proliferated to form colonies of myoblasts. A rare few of these cells have down-regulated MyoD and up-regulated Pax7 (arrow) thus signalling a return to quiescence. (B) Representative image of a single fibre fixed after 72 hours in culture and immunostained for MyoD and myogenin expression. The majority of cells are in the proliferative phase and are MyoD+ myogenin-, but some have begun to differentiate, shown by the up-regulation of myogenin (arrows). (C) shows the number of satellite cells/fibre after 24, 48 and 72 hours in suspension culture in male and female 2 week, 3 month, 1 year and 1.5 year old mice. There was a significant difference between 3 months and 1 year (p = 0.05) and between 1 year and 2.5 years (p = 0.001) after 72 hours in culture. There was no difference between males and females at any time point. (D) shows the percentage of myoblasts either Pax7+ (blue) only, MyoD+ (red) only, or co-expressing Pax7 and MyoD (green) on single fibres from male and female 2 week, 3 month, 1 year and 2.5 year old mice fixed immediately after isolation (i) and after 72 hours in culture (ii). Scale bar 50 µm.

To assess the functionality of satellite cells from our different populations, we analysed single fibres for satellite cell numbers and expression of Pax7, MyoD (Fig. 2A) and myogenin (Fig. 2B) immediately after isolation or after 24, 48 and 72 hours in culture. Twenty fibres were analysed per mouse from at least three mice per condition.

After 72 hours in culture, 3 month old fibres had significantly more satellite cells per fibre than 1 year and 2.5 year old fibres (Fig. 2C). However this can be accounted for by the greater starting number of satellite cells/fibre. It is therefore more meaningful to analyse the number of population doublings. 3 month old satellite cells undergo ∼2 population doublings between 24 and 72 hours in culture. Aged satellite cells have a slower doubling time, as they have undergone only 1 population doubling within the same time period (Fig. 2C).

To observe satellite cell self-renewal, we stained fibres fixed after 72 hours in culture for the expression of Pax7 and MyoD (Fig. 2Dii). In accordance with previous data [10] we observe that self-renewing satellite cells represent a smaller fraction of the satellite cell population in aged compared to adult mice.

In single fibres from 2 week old mice analysed immediately after isolation, the majority (91% males, 92% females) of satellite cells were shown to co-express Pax7 and MyoD (Fig. 2Di) and are thus actively proliferating. This is to be expected from a fibre that, within the next 10 weeks, will almost double its number of myonuclei (Fig. 1C). Intriguingly, however, not all satellite cells are recruited into this growth program. We observed a rare few quiescent satellite cells, MyoD- Pax7+ (Fig. 2Dii). It was not possible to assess the fate of these cells on a single fibre in culture, as 2 week old fibres could rarely be cultured successfully for any length of time. Fibres that could be analysed had no associated satellite cells by 48 hours. We hypothesise that this is due to changes in the basal lamina between 2 week and 3 month old mice. Fibres of 2 week old mice are more fragile and the basal lamina more easily damaged, such that the satellite cells may not remain associated with the fibre and/or the fibre contracts.

### Myonuclei to cytoplasm ratio is a function of developmental stage

**Figure 3:**
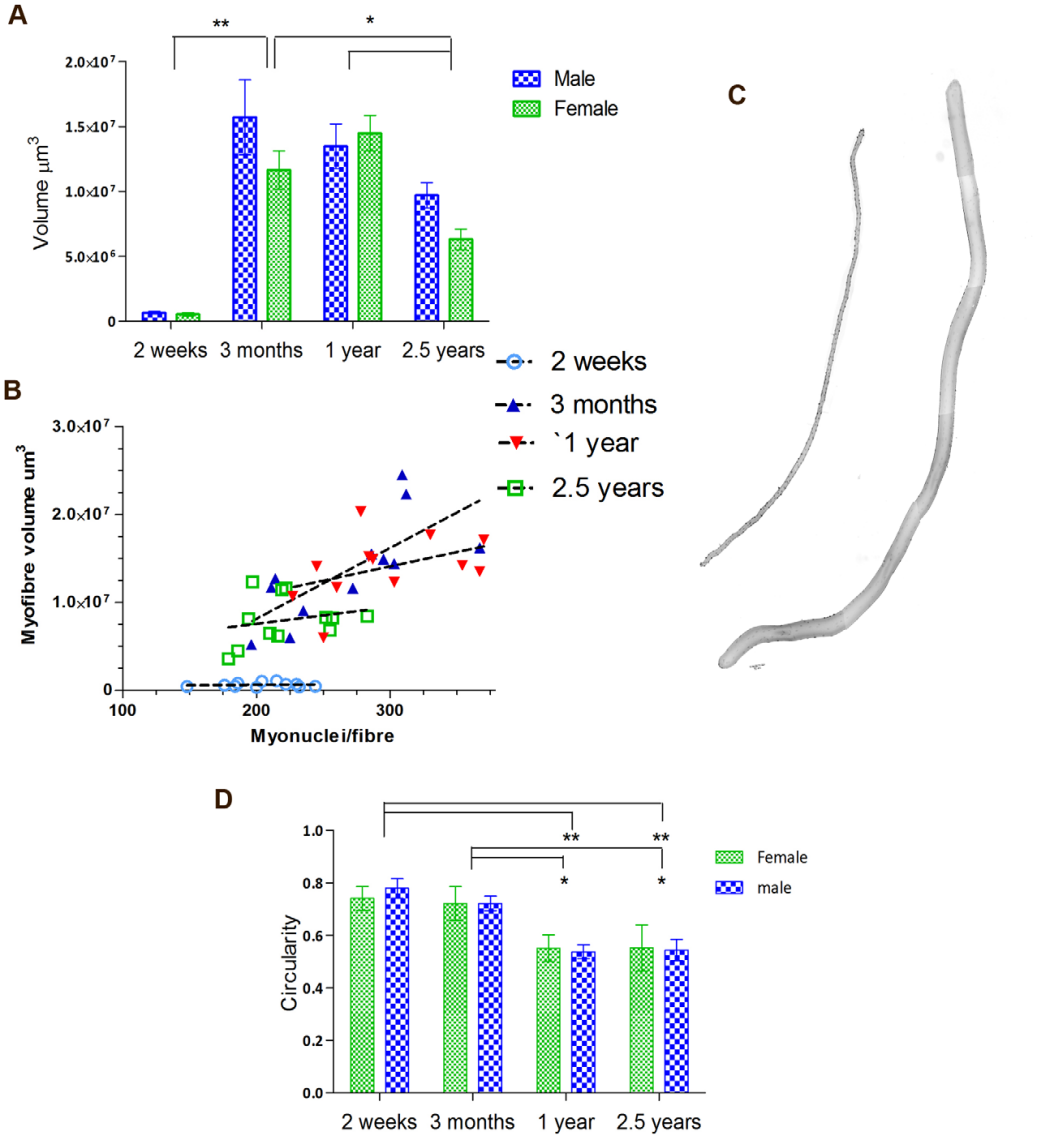
Analysis of the myonuclei and myofibre volume of isolated EDL single fibres. (A) shows changes in myofiber volume across age groups. Volume increases significantly between 2 weeks and 3 months of age (p<.001), and decreases between 1 year and 2.5 years (p<.001). There is no difference in fibre volume between males and females at any age. (B) The relationship between the number of myonuclei per fibre and fibre volume. Pearson correlation coefficients reveal a positive correlation at 3 months and 1 year of age(r = .77 p = .013, r = .58, p = .045 respectively), but no relationship at 2 weeks or 2.5 years of age (r = .23 p = .51, r = .29 p = .35 respectively). (C) Representative images of EDL myofibres stained with DAPI at 2 weeks and 3 months of age. Multiple images taken at 10× magnification have been combined to demonstrate the full length of the myofiber. (D) Two way analysis of variance shows a significant effect of age (p<.0001) but no effect of gender on myonuclear shape. Post hoc tests show myonuclei become less circular between 3 months and 1 year of age (p<.001).

Next, we set out to investigate if the observed changes in myonuclear number are related to changes in myofibre size. The myonuclear domain hypothesis of muscle fibre growth states that the nucleus to cytoplasm ratio remains constant, hence any increase in muscle size must occur via the addition of new myonuclei from the proliferation of satellite cells. Surprisingly, we found no difference in the volume of EDL fibres between males and females within any age group. We observed an increase in myofibre volume between 2 weeks and 3 months of age (p = <0.001) (Fig. 3A) and a decrease in fibre volume between 1 year and 2.5 years (p<0.001). These significant changes were concomitant with the changes in myonuclear number (Fig. 1B) that are predicted by the myonuclear domain hypothesis.

If fibre growth is inextricably linked to myonuclear addition, and thus satellite cell activity, then this relationship ought to be observed between fibres of different sizes within age groups. We analysed Pearson correlation coefficients between the number of myonuclei per fibre and fibre volume within our four age group categories (Fig. 3B). There was an overall positive correlation between myonuclear number and myofibre volume. However this relationship did not exist within all age groups. We observed a significant positive correlation between myonuclear number and fibre size in adult mice (3 months r = .77 p = 0.013, 1 year r = .58, p = 0.045 respectively). However, at 2 weeks and 2.5 years of age this relationship was not observed. Results show that myonuclear domain is established between 2 weeks and 3 months of age and remains fairly constant for adult life. In old age we see an overall decrease in myonuclear number (Fig. 1C) and volume (Fig. 3A) and a breakdown in the relationship between these two measures (Fig. 3B).

Whilst quantifying changes in myonuclear numbers, it was observed that myonuclei have distinctive morphologies according to age (Fig. 1C). At 2 weeks of age, myonuclei are round in shape and appear to have tightly compact chromatin, whilst at 2.5 years nuclei are greatly elongated and chromatin appears less dense. A two way ANOVA showed a significant effect of age on circularity (f = 40.79, p = <0.0001). Post hoc T-tests demonstrate that myonuclei from 2 week and 3 month old mice were significantly more circular than 1 year or 2.5 year old mice (p<0.001) (Fig. 3D).

### Host or donor sex does not affect donor satellite cell contribution to muscle regeneration after transplantation

Thus far, single fibre analyses represent myofibre and satellite cell dynamics within a sedentary model where satellite cells are not challenged beyond the requirements of normal growth. It is possible that male and female satellite cells have functional differences with regards to their ability to survive transplantation and regenerate muscle after severe injury. The significantly greater number of satellite cells found in male compared to female mice may also suggest that male donor mice provide a greater pool of satellite cells and will therefore produce greater numbers of donor derived fibres, making them a preferable choice of donors in current models of satellite cell transplantation.

**Figure 4:**
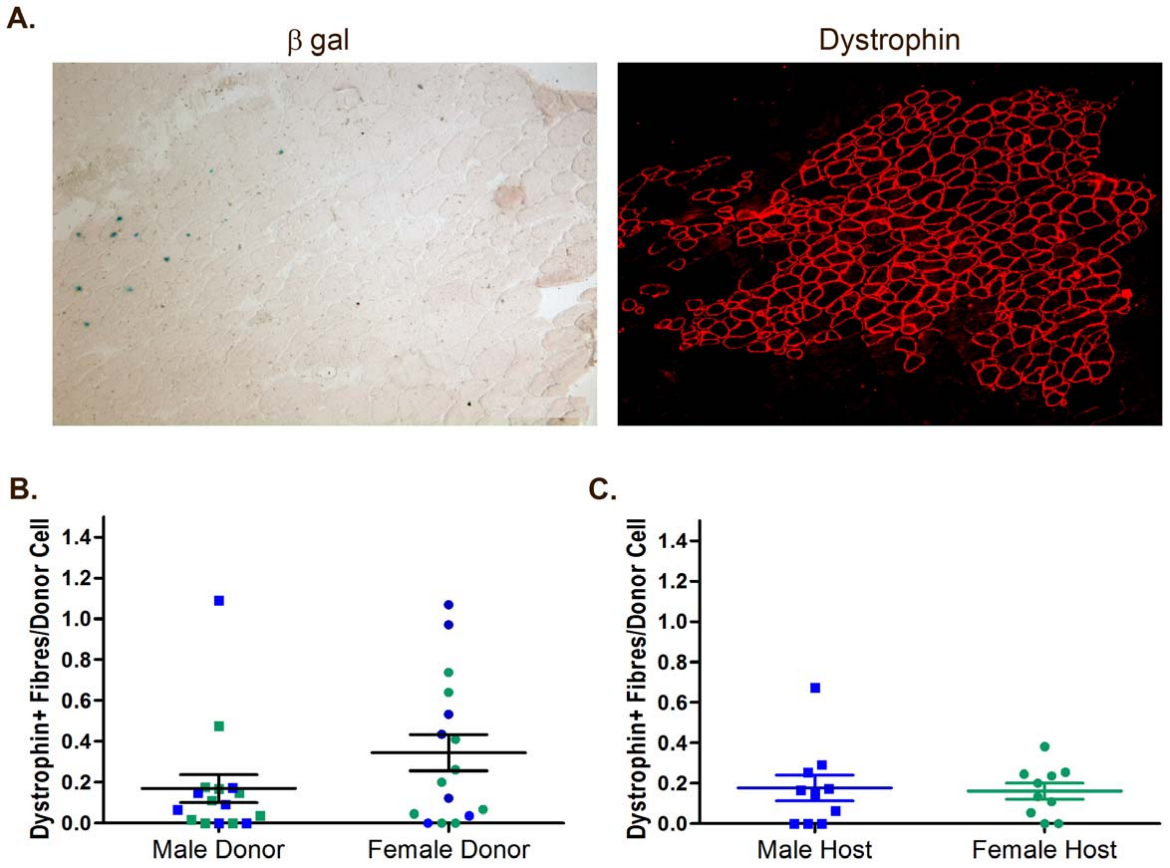
Satellite cells from male and female 3F-*nLacZ*-2E mice show similar contribution to host muscle regeneration. (A) Representative image of donor derived fibres in *mdx*-nude TA muscle sections. X-gal staining shows the presence of donor-derived fibres co-localised with dystrophin positive fibres. Scale bar 50 µm. (B) Donor cells were injected into 4 week old *mdx*-nude mice TA muscles. TA muscles were harvested 4 weeks after cell injection and the number of dystrophin positive fibres quantified. Graph shows no significant difference in the amount of donor muscle produced per injected between male and female 3F-*nLacZ*-2E donors. Blue data points indicate a male host, green indicates a female host. (C) shows no significant difference in the number of dystrophin positive fibres per injected cell between male and female *mdx*-nude hosts injected with satellite cells from male 3F-*nLacZ*-2E donors.

Satellite cells were harvested from freshly isolated male or female 3F-*nLacZ*-2E EDL single fibres. Satellite cells stripped from approximately 100 male or female fibres were injected into contralateral *mdx*-nude *tibialis anterior* (TA) muscles that had been irradiated 3 days before grafting [7], [9], [10], [52]. Muscles were analysed four weeks later for donor contribution to muscle regeneration (Fig. 4A). Despite the differences in the number of satellite cells injected between male and female donors (∼5.5 and ∼3.9 satellite cells per male and female fibre respectively see Fig. 1B), we found no difference in the number of dystrophin positive fibres in host muscles injected with satellite cells derived from male or female donors. Data are presented as the number of dystrophin positive fibres normalised for the number of cells injected (Fig. 4B). A one way ANOVA shows no significant effects of sex of donor and no interaction between the sex of the host and the sex of the donor (f = 1.31 p = 0.29). The number of dystrophin positive fibres formed after transplantation therefore does not correspond in a linear fashion to the number of cells injected.

Our experiments have demonstrated that there is no intrinsic difference between male and female satellite cells in their ability to regenerate host muscle after transplantation. However, it is possible that sex differences in muscle regeneration may occur, and are governed by environmental, rather than via satellite cell intrinsic mediators e.g. circulating androgens. To investigate this, we isolated satellite cells from 3 month old male 3F-*nLacZ*-2E mice and injected them into the TA muscles of male and female *mdx*-nude hosts. We found no difference between the number of donor-derived fibres at 4 weeks post injection between male and female hosts (Fig. 4C). Together these data demonstrate that in our model of satellite cell transplantation, donor derived muscle formation is not altered either by the sex of the host or the sex of the donor.

### Satellite cells from growing and adult mice show comparable contribution to host muscle regeneration after transplantation

*In vitro* analysis of single fibres showed that the age at which the fibre is isolated has the greatest effect on all variables measured. However, previous research has shown that aged and adult donor satellite cells regenerate host muscle equally well [10]. The regenerative capacity of satellite cells isolated from fibres derived from muscles that are actively growing is unknown.

**Figure 5:**
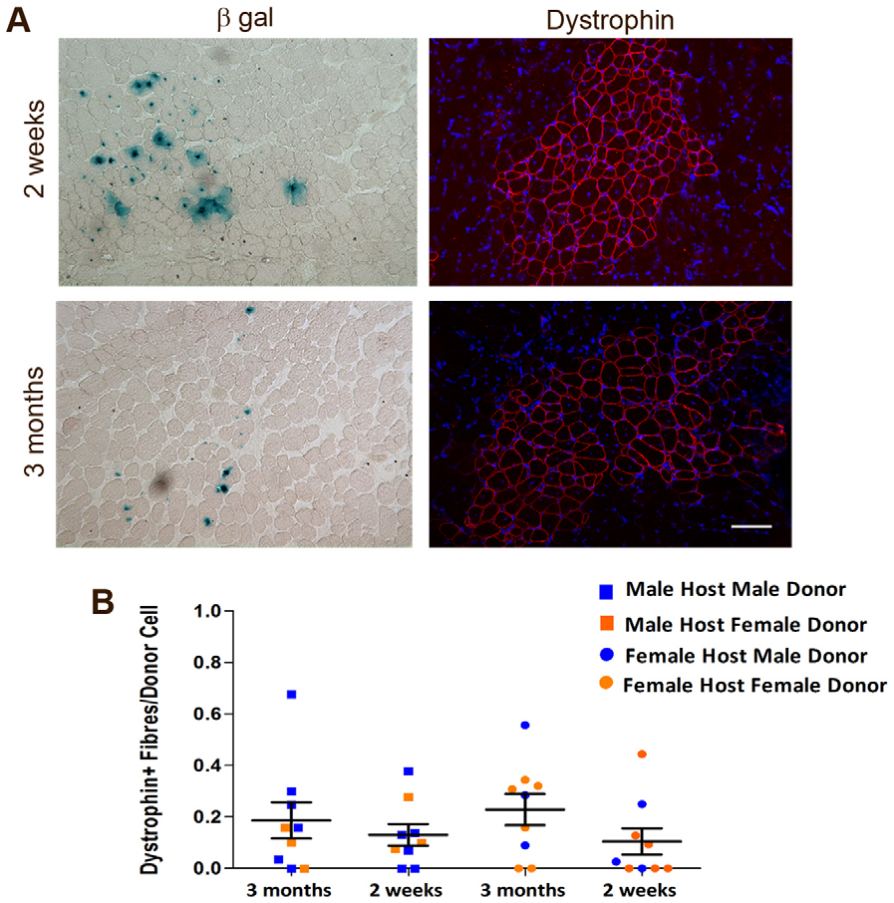
3 month and 2 week old donor mice show similar contribution to host muscle regeneration. Donor satellite cells were injected into 4 week old *mdx*-nude mice TA muscles which were harvested 4 weeks later. Donor contribution to muscle regeneration was quantified by counting the number of dystrophin positive fibres and normalizing for the number of cells injected. (A) Representative sections of *mdx*-nude host TA muscles grafted with donor satellite cells 4 weeks previously. Donor contribution to muscle is shown by β-gal and dystrophin expression. Scale bar 50 µm. (B) There was no difference in the number of donor-derived fibres per injected cell between muscles grafted with cells from 3 month old or 2 week old 3F-*nLacZ*-2E donors either in male or female *mdx*-nude hosts.

We isolated satellite cells from EDL muscle fibres of male 2 week and 3 month old 3F-*nLacZ*-2E mice and injected cells from approximately 100 stripped fibres per donor muscle into the TA muscles of male and female *mdx*-nude hosts. We therefore injected approximately 850 satellite cells from 2 week old donors and 550 satellite cells from 3 month old donors into host muscles. Despite these differences in injected cell numbers, and the differences in proliferative state and mitotic age of the cells, we observe no difference in the number of dystrophin positive fibres in TA muscles grafted with 3 month old or 2 week old satellite cells (Fig. 5A and B). A two way ANOVA showed no effect either of the sex of the host, the sex of the donor and no interaction between 2 week old, 3 month old donors with the sex of either donor or host (p = 0.1) on the number of dystrophin positive fibres per donor cell injected. These data strengthen our conclusion that donor derived muscle formation post transplantation is not the cumulative product of all cells transplanted. At best it seems there is a loose relationship between the number of satellite cells transplanted and the subsequent number of dystrophin positive fibres observed.

## Discussion

These data represent a comprehensive investigation of the satellite cell population, its relationship to myonuclei and its regenerative ability after transplantation across the life span of male and female mice. We show that a small percentage of satellite cells in growing muscle are not actively contributing to myonuclear addition. The satellite cell population changes in abundance as a function of age and sex, yet these changes do not relate to regenerative capacity post transplantation. We hypothesise that the satellite cells that are responsible for host muscle regeneration after transplantation are a distinct population from the more numerous satellite cells responsible for muscle growth and maintenance in situ.

*In vitro* studies of mouse EDL single fibres show that adult males have significantly more satellite cells per fibre than adult females (Fig. 1A). This is in accordance with previous data showing males have more satellite cells per fibre in mice ranging from 3 to 8 months of age [48]. Here we show that sex differences in satellite cell number can only be observed in adult mice (Fig. 1A). The time course of satellite cell population divergence between males and females suggests that androgens may be significant mediators of satellite cell number. Androgen levels peak during puberty, between days 30 and 35 in C57BL/6 mice [53] and decrease with old age [54]. This is parallel to the changes observed in satellite cell numbers in mice with increasing age. Previous data have shown that testosterone can increase satellite cell number and satellite cell proliferation [14], [15], [17]. We show no difference in donor contribution to host muscle regeneration after satellite cell transplantation into male and female *mdx*-nude host mice. From this we conclude that, in our model, although androgens may mediate satellite cell numbers, exposure to a male or female host environment and thus circulating androgens does not mediate satellite cell survival or proliferation after transplantation.

There is research to suggest that male satellite cells have a greater proliferative rate and express higher levels of myogenic regulatory factors ex-vivo than female satellite cells [18]. Here we studied satellite cells in an *in vitro* system that maintains satellite cell myofibre interactions, thus keeping satellite cells within an environment that mimics that seen *in vivo*. This system also allows the analysis of satellite cells at the individual cell level, rather than averaging expression profiles across the population. Our data show a comparable rate of self-renewal, and similar activation and proliferation profiles, in male and female satellite cell populations. There were greater numbers of myoblasts in male mice after 72 hours in culture (Fig. 2C), however this is accounted for by the larger starting number and no difference was observed in population doubling times.

Growing mice have significantly greater numbers of satellite cells than adult mice (Fig. 1A). Presumably satellite cells are lost as the mouse progresses into adulthood because they undergo terminal differentiation to produce the large increase in myonuclear number (Fig. 1B) [4]. This is supported by previous data showing that the rapid increase in myofibre cross sectional area in mice between postnatal day 3 and postnatal day 21 is accompanied by a 5 fold increase in myonuclear number and a decrease in satellite cell number [22]. To facilitate this rapid increase in myonuclear number, satellite cells must undergo many rounds of cell division. However, growing muscle does not recruit all of its satellite cells into this growth programme. At postnatal day 14 we observe that 3% of male and 4% of female satellite cells are quiescent.

Despite differences in number, proliferative state, mitotic age and genetic requirements between adult and postnatal day 14 mice, there is no difference in regenerative efficiency post transplantation. When engrafted into *mdx*-nude hosts, the absolute number of satellite cells does not have a linear relationship with the amount of muscle that can be regenerated from these cells. The number of dystrophin positive fibres obtained four weeks post transplantation was independent of the sex or the age of the donor mice. Together these data suggest that despite considerable variance in the absolute number of satellite cells, the number of cells that can contribute to muscle regeneration post engraftment is small and remains constant. It is possible that those cells observed to be quiescent on single fibres of postnatal day 14 mice are the same small population of satellite cells that are able to contribute to muscle regeneration post transplantation.

It is well established that satellite cells are a heterogeneous population and that those that are able to contribute to host muscle regeneration after transplantation are rare. In the mouse, individual EDL derived myofibres engrafted into the irradiated TA of host mice, show donor-derived fibres in just 1 out of every 8 engraftments [7]. Furthermore, transplantation of individual satellite cells show donor-derived muscle in just 4% of engraftments [8] Much research is aimed at uncovering the mechanism(s) that give(s) rise to satellite cell heterogeneity and biomarkers that could be indicative of a particular satellite cells proliferative/self renewal or engraftment capacity (reviewed [55]). Most recently it has been shown that a subpopulation of satellite cells produce distinct daughter cell fates by asymmetrically segregating template and newly synthesised DNA strands [56]. Our results highlight an important conceptual dissociation between the differing roles of satellite cells across the lifespan. How the functional subpopulations we delineate here relate to the heterogeneity in strand segregation and marker expression detailed elsewhere is an important and exciting question to be determined in future work.

We show that adult myofibres maintain a constant ratio between the number of myonuclei per fibre and the fibre's cytoplasmic volume (Fig. 3C). This is predicted by the myonuclear domain hypothesis, which states that each myonucleus has a restricted area of cytoplasm over which it presides and that this area remains constant [36]. This is supported by studies showing the prevention of muscle hypertrophy after the supposed ablation of satellite cell activity by exposure to high doses of gamma radiation [32]–[34]. There is some research to show that changes in muscle size can occur without satellite cell activity [37]–[39]. Most recently, using the diphtheria toxin receptor under control of the Pax7 locus, McCarthy et al. show that conditional ablation of Pax7 expressing cells does not prevent muscle hypertrophy in response to synergist ablation [35]. However, it must also be noted that in this model the TA muscle was seen to have a significant reduction in mass 2 weeks after satellite cells were incapacitated, which was further exacerbated at 8 weeks. Thus, it seems that satellite cell activity is important in routine muscle maintenance. Furthermore, when satellite cells are functional, synergist ablation and consequent muscle hypertrophy is accompanied by the addition of new myonuclei and new myofibres [57], [58].

In accordance with previous studies [7], [10], [48], [49], our results show that satellite cell number declines steadily with increasing age (Fig. 1A). This decline in satellite cell number is accompanied by a decrease in myonuclear number, myofibre atrophy, and a failure to maintain a myonucleus to cytoplasm ratio (Fig. 1B, Fig. 3B,C). Together these findings are indicative of a role for satellite cells in muscle maintenance throughout life and that the impairment in satellite cell function with old age contributes to sarcopenia. This is supported by analyses of myoblasts on isolated single fibres. After 72 hours in culture we found fewer fibre-associated myoblasts, and perhaps more interestingly, a smaller percentage of these myoblasts returning to quiescence, in aged compared to adult fibres (Fig. 2D).

It is possible that a diminished amount of self-renewal is the cause of the reduced number of satellite cells observed in freshly isolated aged fibres. Despite a diminished ability to maintain muscle mass and satellite cell numbers in situ, previous work has shown that satellite cells from aged donors regenerate host muscle with similar efficiency as young donors in our model of transplantation [10]. These data stress the importance of a conceptual dissociation between satellite cells for muscle growth and routine muscle maintenance and satellite cells for muscle regeneration after injury.

Here, satellite cell populations have been studied across the life span of an animal that is largely sedentary. This has enabled us to draw conclusions about muscle maintenance and homeostasis and is arguably a good model for 21^st^ century humans. Yet it remains possible that muscle fibre hypertrophy in response to exercise works via different mechanisms. However, mice lacking functional Pax7 expressing cells show a significant decrease in muscle mass and dramatic increase in fibrosis after strenuous exercise, compared to exercised wild type and unexercised controls [1]. This indicates that hypertrophy in response to exercise, as with normal muscle growth, is usually associated with satellite cell activity.

We suggest there are at least two distinct satellite cell populations. The first population is responsible for myonuclei addition during growth and general muscle maintenance throughout life. These satellite cells are present in greater numbers in growing muscle, are diminished with age, and are more numerous in adult males compared to females. The second population is formed by those satellite cells that are activated by severe muscle injury and survive transplantation. These cells are present in similar numbers from birth to old age and do not differ between males and females.

## Materials and Methods

### Ethics Statement

Mice were bred, and experimental procedures were carried out in the Biological Services Unit, of University College London, Institute of Child Health, in accordance with the Animals (Scientific Procedures) Act 1986. Experiments were carried out under Home Office licence.

### Single myofibre isolation and culture

2 week, 3 month, 1 year and 2.5 year old male and female C57BL/6 mice were killed by cervical dislocation. *Extensor digitorum longus* (EDL) muscles were isolated from tendon to tendon under microscopic observation as previously described [59]. EDL muscles were digested in 2% collagenase type I (Sigma)/Dulbecco's modified Eagles medium (DMEM; Gibco) at 35°C for 60 (2 week old mice), 70 (3month/1 year old mice) or 90 (2.5 year old mice) minutes. Muscle fibres were serially washed with modified glass Pasteur pipettes to remove cell contaminants and debris. Single fibres were either fixed immediately in 4% paraformaldehyde (PFA) in a 2 ml Eppendorf tube or placed in a horse serum coated dish (approx. 25 fibres/dish) of warmed plating medium (10% horse serum (Gibco), DMEM, 0.005% chick embryo extract, 4 mM L-glutamine (Sigma), 1% penicillin and streptomycin antibiotics) and incubated at 37°C for up to 96 hours.

### Single fibre immunohistochemistry

Single fibres were fixed in 4% paraformaldehyde, permeabilized with 0.5% Triton X-100 (Sigma) and blocked with 10% goat serum. Fibres were incubated overnight at 4°C with primary antibodies: Pax7 (DHSB, mouse monoclonal), MyoD (Santa Cruz, rabbit polyclonal), MyoD (Mouse monoclonal, Dako) and myogenin (Santa Cruz, rabbit polyclonal). Fibres were washed in PBS before being incubated with the appropriate Alexa-Fluor secondary antibody. Fibres were individually placed onto a glass microscope slide using a heat polished glass Pasteur pipette.

### Satellite Cell Grafting

3 days prior to engraftment, 3–4 week old *mdx*-nude mice were anaesthetised with Hypnorm (Janssen: fentanyl citrate, final concentration 0.79 mg/mL: fluanisone, final concentration 2.5 mg/mL), and Hypnovel (Roche: midazolam, 1.25 mg) in distilled H_2_O injected subcutaneously. Mouse hind limbs were irradiated in a Gamma service Medical GmbH Cs-137 irradiator.

On the day of engraftment, single myofibres from donor mice were isolated as described above and satellite cells were stripped from fibres as previously described [9].

Irradiated host *mdx*-nude mice were anaesthetised with isoflourane. Satellite cells were drawn into a fine glass needle and injected into the *tibialis anterior* (TA) muscle under microscopic observation. Injected volume was no greater than 10 µl.

### Histological Analysis of Grafted Muscles

4 weeks post engraftment, host mice were killed by cervical dislocation and the TA muscles removed. TA muscles were frozen in isopentane cooled in liquid nitrogen. Muscles were kept at −80 until cryosectioning. 7 µm transverse sections were collected at 100 µm intervals from the whole muscle. Sections were air-dried for at least 20 mins before being stored at −80 until use.

Cryosections were rehydrated with PBS and X-gal stained as described previously [60]. Sections serial to those with X-gal +ve donor-derived nuclei were analysed for dystrophin expression. Sections were blocked with 10% goat serum, and stained with primary rabbit anti-dystrophin (P7) [60], and Alexa Fluor 594-conjugated goat anti-rabbit Ig (Molecular Probes). Sections were mounted on 3-aminopropyltriethoxysilane (APES)-coated slides with fluorescent mounting medium (DAKO) containing DAPI.

### Image capture and quantitative analysis

Fluoresence and brightfield images were taken using a Zeiss Axiophoto microscope (Carl Zeiss, UK) and Metamorph image capture software (Metamorph production, UK). Any further image processing was achieved with ImageJ (rsbweb.nih.gov/ij). Myofibre length and width and myonuclear shape was analysed using ImageJ software. Where s = shape and C = circularity, circularity can be calculated as
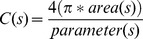
Volume was calculated assuming that the myofibre is cylindrical in shape using
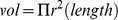
Graphs and images were assembled using Photoshop CS4.
